# Sensitive detection of free bilirubin in blood serum using β-diketone modified europium-doped yttrium oxide nanosheets as a luminescent sensor

**DOI:** 10.1039/c8ra02817f

**Published:** 2018-05-16

**Authors:** Wei Yang, Jinfeng Xia, Guohong Zhou, Danyu Jiang, Qiang Li

**Affiliations:** Department of Chemistry, East China Normal University Shanghai 200062 P. R. China qli@chem.ecnu.edu.cn; Shanghai Institute of Ceramics, Chinese Academy of Sciences Shanghai 200050 P. R. China

## Abstract

Free bilirubin, when present in excess in the human body, can cause a multitude of diseases and disorders and even be fatal; hence, detecting it is of paramount importance. Herein, we report a luminescence quenching-based non-enzymatic method for the convenient, reliable, and rapid detection of free bilirubin in blood serum samples using sensing films (nanosheets/PS, nanosheets-tta/PS, and nanosheets-dbt/PS) as luminescent sensors. The luminescence intensity of the sensing films is linearly related to the free bilirubin concentration. Nanosheets-tta/PS demonstrated excellent sensing properties for the sensitive and reliable detection of free bilirubin in the range of 0.0–60.0 μM with a correlation coefficient of 0.9915, as compared to nanosheets/PS or nanosheets-dbt/PS. The limit of detection for the determination of free bilirubin was 41 nM. This method can be used to design a sensor-based test spot as a medical detection device for the visual detection of free bilirubin.

## Abstract 2

Free bilirubin, when present in excess in the human body, can cause a multitude of diseases and disorders and even be fatal; hence, detecting it is of paramount importance.

## Graphical abstract

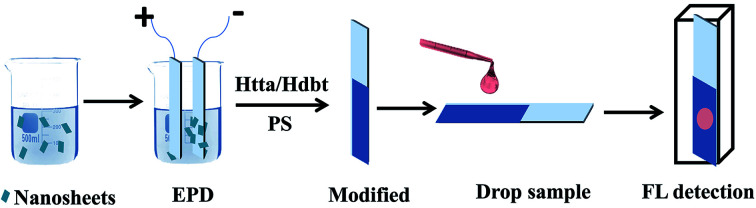

## Introduction

Bilirubin (BR) is a yellow metabolic breakdown product of normal blood and has important biological and diagnostic significance.^1,2^ Free BR, also known as BR IX, has a lipophilic nature and plays a significant role in the tissue uptake and toxicity of BR.^3^ The normal concentration level of BR IX in human serum is less than 25 μmol L^−1^, which increases to >50 μmol L^−1^ in a jaundice-infected individual.^4–6^ In addition to jaundice, excess BR IX can cause hepatitis, mental disorders, cerebral palsy, brain damage, and even death.^7–9^ Therefore, precise determination of the concentration of free BR is extremely important.

To date, there have been many analytical methods for determining the BR IX concentration in serum samples; these include modified diazo methods, oxidation methods, bio-enzymatic methods,^10^ separation-based methods,^11^ electrochemical biosensing,^12,13^ and fluorescence measurements.^14^ In most of these methods, the sample needs to be pretreated; moreover, the process of detection is complicated and indirect. The accuracy of detection using a bio-enzymatic method depends on a series of environmental conditions such as pH and temperature, in addition to the inconvenient processes of extraction and storage of bio-enzymes. Electrochemical sensor electrodes are easily disturbed by biological media. Therefore, there is urgent need to explore and develop a new non-enzymatic method for the direct, rapid, reliable, and visual detection of free BR in serum samples.

Fluorometric methods seem to be the most suitable means of detecting BR IX. Lanthanides have advantages such as high fluorescence quantum yields, large Stokes' shifts, strong luminescences, narrow luminescence bands, and long fluorescence lifetimes.^15–17^ In recent years, lanthanides have increasingly been used for biological detection.^17,18^

This paper describes a BR IX sensor based on Eu(iii)-doped nanosheets. These nanosheets have all the advantages of Eu(iii) complexes, and possess unique characteristics due to unusual structural features such as excellent two-dimensional anisotropy. More importantly, these Eu(iii)-doped yttrium oxide nanosheets overcome the shortcomings of Eu(iii) complexes, and exhibit enhanced luminescence and higher stability.^19^ Furthermore, the above nanosheets can be used to prepare fast-response thin-film planar optodes and optical fibers.

The Eu(iii)-doped yttrium oxide nanosheet sensing films, after their successful fabrication, were modified with 2-thenoyltrifluoroacetonate (Htta) or 2-acetylbenzothiophenetrifluoroacetone (Hdbt) and coated with polystyrene (PS), in order to further improve their fluorescence intensity, monochromaticity, BR IX sensitivity, and hydrophobicity.

## Material and methods

### Chemicals, reagents, and apparatus

Y_2_O_3_, Eu_2_O_3_, Htta, and Hdbt were obtained from J&K Chemicals. BR IX was obtained from Sigma-Aldrich (USA). All chemicals and reagents in this study were of analytical grade and were used directly without further purification. Luminescence spectra were recorded on a FLS-980 spectrofluorometer (Edinburgh Instruments, UK) equipped with a quartz cuvette (1.0 cm × 1.0 cm) using 1 nm-wide slits for excitation and emission. The UV-vis absorption spectra were recorded on an Agilent Cary UV-8000 spectrophotometer (Tianmei, China). The photoluminescence decays of the sensing films were recorded on an FLS-980 spectrofluorometer (Edinburgh Instruments, UK) equipped with 356 and 367 nm lasers.

### Synthesis of Eu(iii)-doped yttrium oxide nanosheet film

**Fig. 1:**
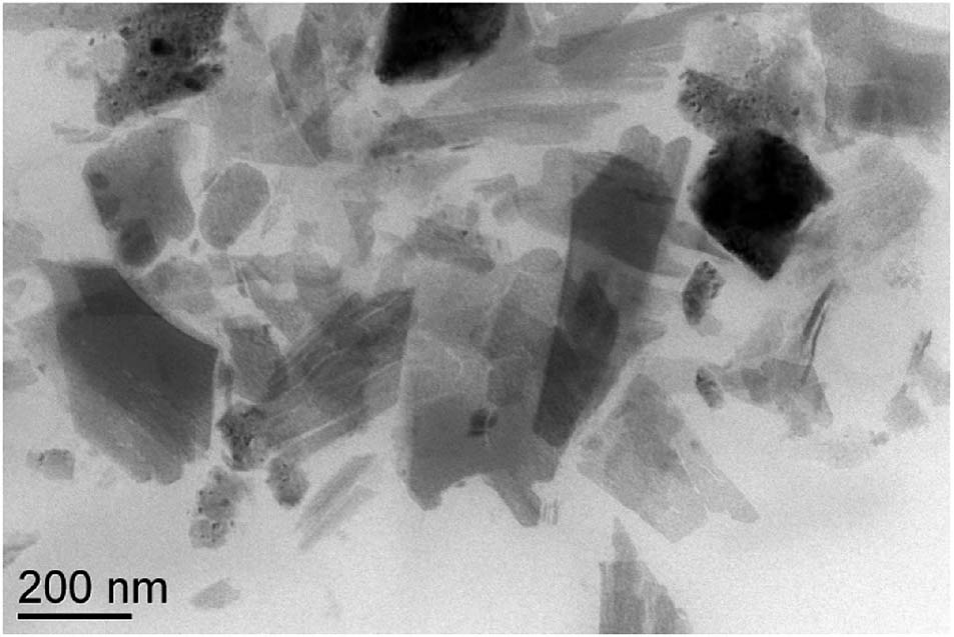
Transmission electron microscopy (TEM) image of Eu(iii)-doped oxide nanosheets.

Eu(iii)-doped oxide nanosheet sols were manufactured according to a procedure described previously.^19^ The as-received nanosheets had lateral dimensions of several hundreds of nanometers. Transmission electron microscopy (TEM) was used to confirm the ultra-thin properties of the nanosheets (Fig. 1).

**Fig. 2:**
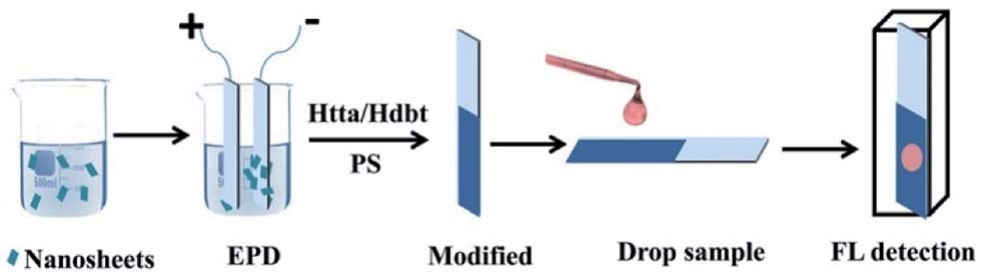
Processes of fabrication and sample detection using the nanosheets-based sensing film.

The preparation process of the sensing films and detection process of BR IX are shown in Fig. 2. Firstly, the nanosheets obtained above were uniformly dispersed in *n*-butanol (100 mL) by 40 min sonication. Secondly, the positively charged nanosheets were electrophoretically deposited (EPD) onto conductive glass (FTO substrate) at 60 V for 10 min, after which a uniform film was obtained. Thirdly, the film was modified with ligands, by immersing it in an ethanol solution containing Htta (0.01 g) or Hdbt (0.01 g) for 5 min, then dried in air. The above coating process was repeated thrice to obtain a luminescent film of Htta/Hdbt-modified nanosheets. Finally, the BR sensing film was obtained by dip-coating it in dry CH_2_Cl_2_ (10 mL) containing PS (0.1 g) at a rate of 2500 μm s^−1^.

Fresh human blood samples were collected from healthy volunteers. All experiments were performed in accordance with the Guidelines “Declaration of Helsinki (2002 edition)” and “Measures for Ethical Review of Biomedical Research involving People”, and experiment approved by “the Academic Ethics Committee of East China Normal university”. Informed consents were obtained from human participants of this study. The original content of BR IX in these samples was removed using a reported method.^20–22^ Serum samples containing BR IX (1–200 μM) were equilibrated at room temperature (RT). Thereafter, 30 μL of the test sample was dropped onto the surface of the sensing films, as shown in Fig. 2. The sensing film was placed in a cuvette and the BR IX concentration was detected by a fluorescence spectrophotometer. In another method, the sensing film was placed directly under UV light to evaluate its efficiency as a point-of-care device for visually detecting BR IX.

### Detection of BR IX by three sensing films

**Fig. 3:**
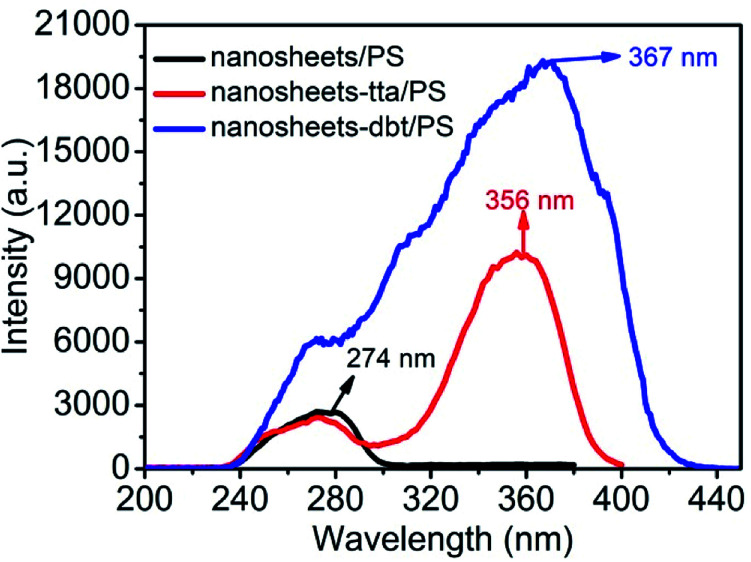
Excitation of nanosheets/PS, nanosheets-tta/PS, and nanosheets-dbt/PS.

Fig. 3 shows the excitation spectra of the nanosheets without and with the ligands (Htta or Hdbt), which were recorded at 274, 356, and 367 nm, for the PS-encapsulated Eu(iii)-doped yttrium oxide nanosheets (nanosheets/PS), PS-encapsulated Htta-modified Eu(iii)-doped yttrium oxide nanosheets, (nanosheets-tta/PS), and PS-encapsulated Hdbt-modified Eu(iii)-doped yttrium oxide nanosheets (nanosheets-dbt/PS), respectively. The luminescence responses toward different concentrations of BR IX were determined in triplicate to provide average values.

## Results and discussion

### Luminescence response of sensing films to BR IX

**Fig. 4:**
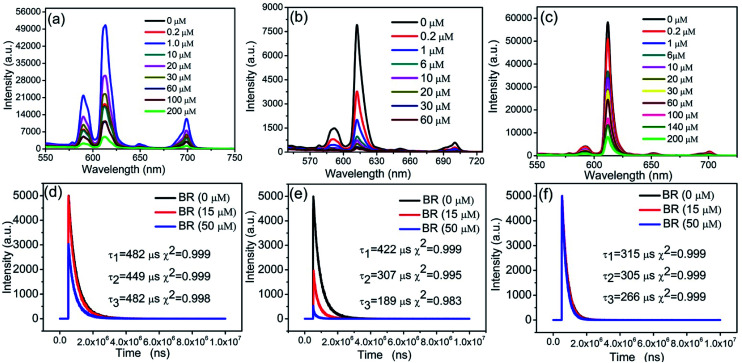
Luminescence emission spectra of nanosheets/PS (a), nanosheets-tta/PS (b), and nanosheets-dbt/PS (c). Fluorescence lifetime of nanosheets/PS (d), nanosheets-tta/PS (e), and nanosheets-dbt/PS (f).

As shown in Fig. 4, nanosheets/PS, nanosheets-tta/PS, and nanosheets-dbt/PS have the same emission maxima at 614 nm, which is the characteristic emission from the ^5^D_0_–^7^F_2_ transition of Eu(iii). Nanosheets/PS, nanosheets-tta/PS, and nanosheets-dbt/PS were excited at 274, 356, and 367 nm, respectively, and the emission peaks were monitored in the 550–750 nm range. As shown in Fig. 4(b) and (c), the luminescence intensity of nanosheets-tta/PS and nanosheets-dbt/PS gradually decreases with increasing BR IX concentration. Only when the BR IX concentration is greater than 20 μM, does the luminescence intensity of nanosheets/PS decrease with increasing BR IX concentration, which is not suitable for detecting the BR IX concentration in the normal range (6.0–17.1 μM). The detection ranges of nanosheets-tta/PS (0.0–60 μM) and nanosheets-dbt/PS (0–200 μM) observed herein are suitable to detect the BR IX concentration in human serum samples. The luminescence intensity of nanosheets-tta/PS is almost completely quenched at a BR IX concentration of 60 μM, (Fig. 4(b)), whereas the nanosheets-dbt/PS retains a strong luminescence intensity even at 200 μM (Fig. 4(c)). An investigation of the effect of BR IX on the fluorescence lifetime of the three sensing films in Fig. 4(d)–(f) revealed that BR IX exhibited no regularity in the fluorescence lifetime of nanosheets/PS, with 15 μM of BR IX having a significant influence, and 50 μM, no influence. The properties of the sensing film containing nanosheets-tta/PS were greatly affected by the BR IX concentration; its fluorescence lifetime decreased with increasing BR IX concentration (0–50 μm). The fluorescence lifetime decayed from 422 μs to 189 μs, with a reasonably high decay efficiency, which further demonstrates that the highly sensitive nanosheets-tta/PS can be used for the detection of BR IX. The fluorescence lifetime of nanosheets-dbt/PS also decreased with the increase in BR IX concentration; its attenuation was greater than nanosheets/PS and less than nanosheets-tta/PS.

### Interactions of BR IX with sensing films

**Fig. 5:**
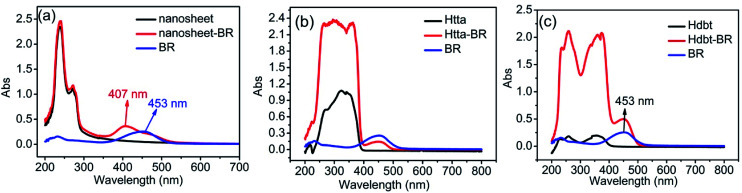
Absorption spectra of nanosheets, nanosheets-BR IX, BR IX (a); Htta, Htta-BR IX, BR IX (b); Hdbt, Hdbt-BR IX, BR IX (c).

There are two possible interactions between BR IX and nanosheets or BR IX and the ligands (Htta, Hdbt). The UV-vis absorption spectra of BR IX, nanosheets, Htta, and Hdbt are shown in Fig. 5. It can be seen that a narrow absorption peak of rare earth ion (Re(iii)) appears at 238 nm in Fig. 5(a), which is contributed to the Y^3+^–O^2−^ charge transfer.^23^ The band at 271 nm in Fig. 5(a) was attributed to the Eu^3+^–O^2−^ charge transfer,^24^ The bands appearing in the ligands at 200–300 nm in Fig. 5(b) and (c) are due to n → π* transition of keto–enol tautomerism.^25^ The broad bands in the 300–400 nm range for the ligands are due to the singlet–singlet π → π* transition of enol absorptions.^26,27^ The maximum-absorption band for BR IX at around 450 nm is due to the superposition of two electronic transitions in BR IX, near 480 nm and around 430 nm.^28^ The absorption spectra of BR IX shift from 453 nm to 407 nm after adding the nanosheets, which reveals that BR IX coordinates to Re(iii) in the nanosheets, as reported in literature.^29,30^ Comparison of the absorbance spectra of BR IX before and after the addition of ligands (Htta, Hdbt) reveals no shift and no new peak generation. Therefore, BR IX does not coordinate with Htta and Hdbt.

**Fig. 6:**
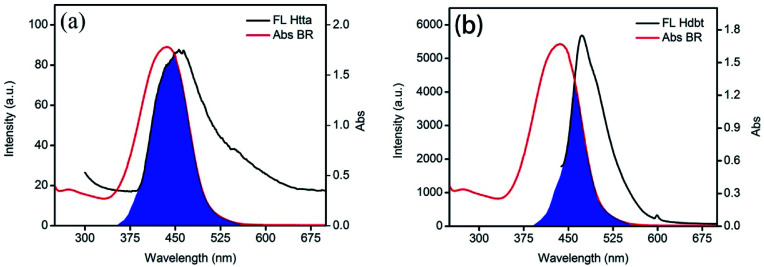
Overlap of the fluorescence emission spectra of Htta (a) and Hdbt (b) with the absorption spectra of BR IX.

Although BR IX and Re can be coordinated, BR IX can be better detected by nanosheets-tta/PS and nanosheets-dbt/PS than nanosheet/PS. As seen in Fig. 6, the emission spectra of Htta (456 nm) and Hdbt (473 nm) overlap with the UV absorption spectrum (453 nm) of BR IX. The overlapping part is marked in blue; it is clear that the overlapping area of Htta and BR IX is larger than that of Hdbt and BR IX. The excitation peak of the nanosheets is located at 274 nm, which has almost no overlap with the excitation peak of BR IX. According to Föster's resonance energy transfer theory (FRET),^31,32^ the larger the overlap area, the better the energy matching, and the more energy the ligands deliver to BR IX. Therefore, Htta can deliver energy to BR IX more efficiently than Hdbt and nanosheets. This result is also consistent with the fluorescence lifetime results in Fig. 4.

The luminescence mechanism of the BR IX-quenching nanosheets-tta/PS and nanosheets-dbt/PS is due to the antenna effect and Föster's nonradiative energy transfer theory. The ligands (Htta, Hdbt) on nanosheets-tta/PS and nanosheets-dbt/PS are coordinated with Eu(iii), respectively, and the absorbed energy is transferred from the ligands to the luminescent center. The characteristic emissions of Eu(iii) then appear. After dropping BR IX on the sensing films, BR IX is coordinated with Re in the nanosheets, and the energy transferred by the ligand matches that absorbed by BR IX. Consequently, the energy delivered by the ligands to the luminescent center is reduced, due to which the luminescence intensity of Eu(iii) also decreases. The quenching process can be expressed by the following equation: [C] + *n*[Q] → [C⋯*n*Q]^33,34^ (C = nanosheets-tta/PS, or nanosheets-dbt/PS).

The energy was transferred from coordinated ligands to *n* equivalents of coordinated BR IX, and BR IX concentration is expressed as [Q]. The luminescence of nanosheets-tta/PS and nanosheets-dbt/PS are quenched as described above. The regression lines of nanosheets-tta/PS and nanosheets-dbt/PS were plotted using the following equations:^20^1
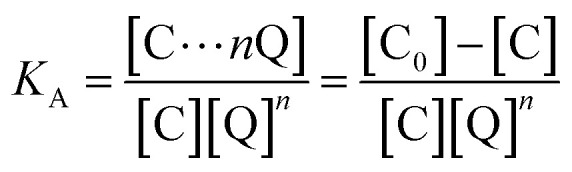
2
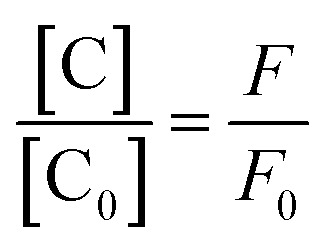
3
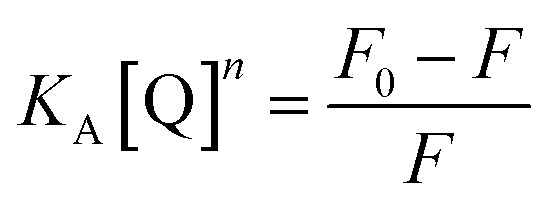
4
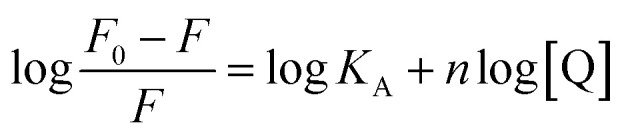
where C and C_0_ are the sensing films with and without BR IX; *F* and *F*_0_ are the luminescence intensities of the sensing films with and without BR IX, respectively.

**Fig. 7:**
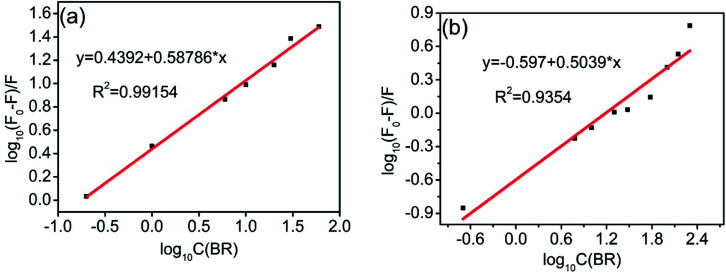
Plot of log[(*F*_0_ − *F*)/*F*] as a function of log[BR IX] nanosheets-tta/PS (a), and nanosheets-dbt/PS (b).

Eqn (4) was used to generate luminescence response curves for the detection of BR IX by the sensing films. As shown in Fig. 7, the logarithm of the luminescence intensity of nanosheets-tta/PS and nanosheets-dbt/PS (log((*F*_0_ − *F*)/*F*)) is proportional to the logarithm of the BR IX concentration (log[BR IX]), in the concentration range of 1–60 μM and 1–200 μM, respectively. The detection limit (*C*_LOD_) is defined by IUPAC and calculated by the formula *C*_LOD_ = 3*S*_b_/*m*.^35,36^ The limits of detection of nanosheets-tta/PS and nanosheets-dbt/PS were 41 nM and 138 nM, respectively. Compared to nanosheets-dbt/PS, nanosheets-tta/PS exhibits better linearity (*R*^2^ = 0.99154) and a lower detection limit. The broad detection range, high detection reliability, and ultra-low detection limit of nanosheets-tta/PS demonstrates its potential use as an excellent visualization sensor of BR IX.

The various methods used for detecting BR IX are listed in Table 1. The molecular imprinting-based method has a wide detection range, but a very high minimum detection limit. The Ru(bipy)_3_^2+^-based fluorescence method^37^ has a sufficiently low detection limit; however, its detection range (33–300 μM) is unsuitable for detecting BR IX, because the normal human bilirubin concentration is lower than 25 μM. The fluorescent protein-based method^38^ and (BOx)-based method^7^ both seem to be the best candidates for BR IX testing; however, they require fluorescent proteins or enzymes, which pose a significant challenge for probe preparation and maintenance. The photoelectrochemical method^39^ has a low detection limit, but a very narrow detection range. The Multiple Organ Failure (MOF)-based fluorescence method^3^ has a wide detection range and a low detection limit. However, the fluorescence intensity of MOF is small, and the preparation of MOF is complicated. Electrochemical biosensing-based method^13^ has a high sensitivity and low detection limit. The bioelectrode was successfully applied to measure the bilirubin content in spiked serum samples. Among the methods listed in Table 1, only the S,N-doped carbon dots-based method^14^ and the method developed in this study can be used to prepare a solid-state sensor. The sensor made from S,N-doped carbon dots has a very high sensitivity; however, its detection range is not suitable to detect BR IX in humans. Nanosheets-tta/PS as a solid-state sensor not only has a low detection limit, but also has a suitable detection range for BR IX detection in humans. It can be employed as a visual inspection instrument in the future.

**Table 1:** Methods for the determination of BR IX

Methods	Linear range (μmol L^−1^)	Detection limit (nmol L^−1^)	Principal part	Correlation coefficient	
Molecular imprinting	1.71–85.51	770	(PHEMATrp) nanofilm (MIP)	*R* ^2^ = 0.98	36
Fluorescent protein-based	0–1197	—	Protein UnaG	*R* ^2^ = 0.956	38
Fluorescence-based method	33–300	52	Ru(bipy)_3_^2+^	*R* ^2^ = 0.998	37
Fluorescence-based method	0.0001–100	5.9 × 10^−3^	MOF	*R* ^2^ = 0.998	3
Photoelectrochemical	0.03–28	1	TiO_2_-polypyrrole	*R* ^2^ = 0.998	39
(BOx)-based method	0–100	4 × 10^3^	BOx enzymes	—	7
Electrochemical-based method	0.2–7	86.32	(HSA)-stabilized Au_18_ nanoclusters	*R* ^2^ = 0.98	13
Fluorescent-based method	0.0002–0.002	0.12	S,N-doped carbon dots	*R* ^2^ = 0.98	14
The developed method	0–60	41	Nanosheets-tta/PS	*R* ^2^ = 0.991	—

**Fig. 8:**
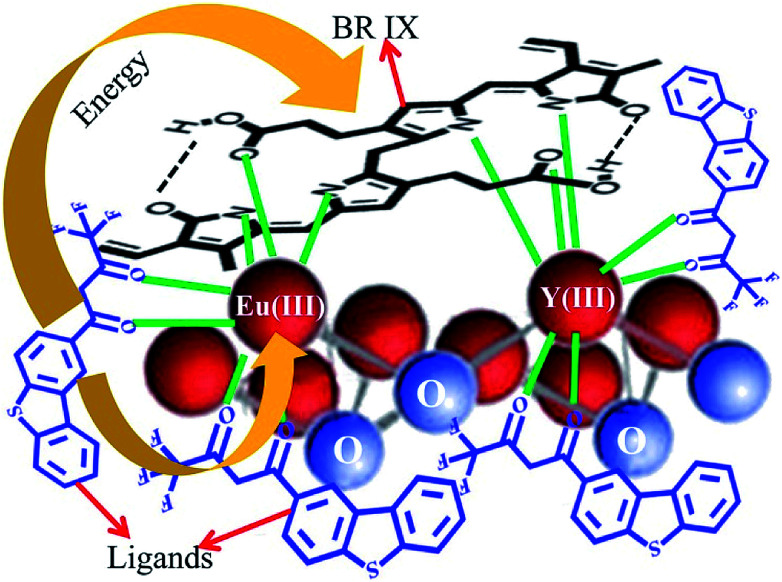
Energy transfer mechanism for luminescence quenching in BR IX-nanosheet-tta/PS and BR IX-nanosheets-dbt/PS.

The number of BR IX molecules coordinated with Eu(iii) has a significant influence on the energy transfer efficiency. The number of BR IX molecules (*n*) coordinated with Eu(iii) onnanosheet-tta/PS and nanosheet-dbt/PS (*n*) is obtained from the slope of their fitted line according to eqn (4), and is 0.58 and 0.50, respectively. The UV absorption spectra in Fig. 5 reveal that the BR IX molecule is coordinated with Eu(iii)/Y(iii). As shown in Fig. 8, the middle club model is the structure of the nanosheet, the black molecular is the molecule of BR IX, and the blue molecules refer to ligands (Htta or Hdbt). One BR IX was coordinated to two Re(iii), one Eu(iii) and one Y(iii), and there are multiple ligands coordinated with each Eu(iii)/Y(iii). When the ligands transfer energy to the luminescent center, the energy transferred by the ligand is absorbed by BR IX, because the energy of the ligand matches that absorbed by BR IX, resulting in the net reduction of the energy obtained from the luminescent center, and thereby causing luminescence quenching.

## Conclusions

We successfully prepared an Eu(iii)-doped yttrium oxide nanosheet film and modified it with Htta/Hdbt and PS to improve the luminescence and service life of the film. The obtained sensing film, nanosheet-tta/PS, could sensitively and reliably detect BR IX in human serum samples. The luminescence intensity of nanosheet-tta/PS was effectively quenched by BR IX *via* a FRET process. Since the rare-earth nanosheet can be deposited by electrophoretic deposition or spin coating, it can be coated on a conductive material or fiber surface to enable the easier detection of BR IX by the sensing film. Nanosheet-tta/PS can thus be used as a film-based sensor in testing for BR IX.

## Conflicts of interest

There are no conflicts to declare.

## Supplementary Material
